# Linear stability and dispersive soliton propagation in nonlinear media subject to parabolic phase modulation

**DOI:** 10.1038/s41598-026-52445-3

**Published:** 2026-05-18

**Authors:** M. Morgan, Hamdy M. Ahmed, M. Sayed, Mahmoud Soliman

**Affiliations:** 1 Department of Physics and Engineering Mathematics, Faculty of Electronic Engineering, Menoufia University, Menouf, 32952 Egypt; 2 Department of Physics and Mathematics Engineering, Faculty of Engineering, German University, Cairo, Egypt; 3 Department of Physics and Engineering Mathematics, Higher Institute of Engineering, El Shorouk Academy, Cairo, Egypt; 4 Department of Physics and Mathematics Engineering, Faculty of Engineering, Ain Shams University, Cairo, Egypt

**Keywords:** Optical solitons, Parabolic self-phase modulation, Linear stability analysis, Improved modified extended tanh-function method, Nonlinear Schrödinger differential equation, Mathematics and computing, Optics and photonics, Physics

## Abstract

This work investigates dispersive optical solitons governed by a perturbed cubic–quartic nonlinear Schrödinger equation with parabolic self-phase modulation, a model of direct relevance to high-capacity fiber-optic systems where simultaneous higher-order dispersion and nonlinear perturbations shape pulse dynamics. The model is physically motivated by fibers with intensity-dependent refractive index profiles, where the interplay between fourth-order chromatic dispersion and parabolic (cubic–quintic) nonlinearity generates wave structures that the standard Kerr approximation cannot capture. To extract exact traveling-wave solutions, we employ the improved modified extended tanh-function method (IMETFM), which is selected for its ability to handle multi-parameter auxiliary equations and yield a wider diversity of solution families than classical expansion methods such as the tanh-function or $G'/G$-expansion approaches, without requiring the integrability of the underlying system. Our analysis produces five families of exact solutions: bright solitons, dark solitons, exponential-type solutions, singular periodic waves, and solutions expressed in terms of Weierstrass elliptic functions. For each family, explicit existence conditions and free-parameter restrictions are stated. The parametric constraints governing solution validity are derived and physically interpreted in terms of the dispersion, nonlinearity, and perturbation coefficients. Graphical representations of the spatial and temporal profiles illustrate the distinct propagation features of each solution type. A linear stability analysis, conducted via perturbation theory, yields an explicit eigenvalue dispersion relation and identifies a critical wavenumber threshold at which modulational instability sets in. The stability criteria provide actionable guidelines for maintaining soliton integrity under weak disturbances in practical optical environments. The results have direct implications for optical fiber communications, ultrafast signal processing, and dispersion-engineered photonic waveguides. The novelty lies in the simultaneous treatment of the parabolic law nonlinearity, fourth-order dispersion, and perturbative effects within a unified algebraic framework, yielding solution families including Weierstrass elliptic solutions that have not previously been reported for this model. Future work will address numerical validation, extension to stochastic and variable-coefficient models, and higher-dimensional soliton dynamics.

## Introduction

Optical solitons propagating in fibers have profoundly reshaped modern telecommunications, making ultra-high-speed signal transmission possible and distortion-free data transmission over long distances. This Extraordinary stability results from a delicate balance between the Kerr nonlinear effect and chromatic dispersion, allowing solitons to preserve their shape and intensity during propagation^1–3^. Such properties underpin modern high-capacity fiber-optic networks, supporting terabit-per-second data rates across transoceanic links, while driving progress in ultrafast lasers, all-optical signal processing, dense wavelength-division multiplexing (WDM), and soliton-based switching technologies^4–6^.

Nonlinear wave equations are central to contemporary physics and engineering, with wide-ranging applications in fluid dynamics (modeling rogue waves and turbulence), plasma physics (wave-particle interactions), and high-bandwidth optical systems^7–9^. The nonlinear Schrödinger equation (NLSE) stands out as a cornerstone model for describing self-sustaining, localized wave packets-solitons that resist dispersive spreading through nonlinear self-focusing or defocusing mechanisms. Recent studies have significantly broadened the landscape of known soliton structures in NLSE-type systems. Controlled fusion and compression dynamics of W-shaped and bright solitons in birefringent optical fibers have been investigated in^10^, demonstrating that Perturbative effects can be harnessed to engineer soliton interactions. Shape-changing soliton formation for a generalized NLSE incorporating third-order dispersion and self-steepening have been reported in^11^, revealing the sensitivity of soliton morphology to higher-order perturbation terms. Chirped dark soliton propagation under simultaneous self-phase modulation and self-steepening for a higher-order NLSE has been studied in^12^, establishing explicit chirp-amplitude relations of direct relevance to dispersion-managed systems. W-shaped chirp-free and chirped bright and dark solitons for the perturbed NLSE in nonlinear optical fibers has been derived in^13^, while dark and singular soliton dynamics in optical fibers have been examined via extended rational sinh- cosh and sine- cosine methods in^14^. W- and M-shaped soliton structures for an eighth-order NLS equation have been reported in^15^, and the deformation of inhomogeneous vector optical rogue waves in variable-coefficient coupled cubic- quintic NLSE with self-steepening has been analyzed in^16^. Collectively, these works underscore the richness of soliton dynamics accessible to higher-order perturbed NLSE models directly motivate the present investigation.

In deterministic optical systems, higher-order dispersion and additional nonlinear perturbations significantly influence soliton dynamics. The perturbed cubic-quartic NLSE incorporating a parabolic law of self-phase modulation provides a realistic framework for capturing these effects in fibers with intensity-dependent phase shifts. This model is physically motivated by fibers with intensity-dependent refractive index profiles, where the interplay between fourth-order chromatic dispersion and parabolic (cubic-quintic) nonlinearity produces wave structures beyond the reach of the standard Kerr approximation^17,18^. Related NLSE frameworks with higher-order dispersion and nonlinearity have been studied in^19–21^, reinforcing the physical relevance and timeliness of the model examined here. The governing deterministic equation is

1 $$\begin{aligned} i \frac{\partial q}{\partial t} + a_1 \frac{\partial ^2 q}{\partial x^2} + a_2 \frac{\partial ^3 q}{\partial x^3} + a_3 \frac{\partial ^4 q}{\partial x^4} + \bigl (b_1 |q|^2 + b_2 |q|^4\bigr ) q =i \alpha \frac{\partial }{\partial x}\bigl (|q|^2 q\bigr )\ + i\beta \, |q|^2 \frac{\partial q}{\partial x} + i\gamma q \frac{\partial }{\partial x}\bigl (|q|^2\bigr )\ \end{aligned}$$

where *q*(*x*, *t*) is the complex envelope function; $a_1$, $a_2$, $a_3$ are the second-, third-, and fourth-order dispersion coefficients, respectively; $b_1$ and $b_2$ are the cubic and quintic self-phase modulation coefficients; and $\alpha$, $\beta$, $\gamma$ represent perturbation terms arising from self-steepening, nonlinear dispersion, and cross-phase modulation effects, respectively. This model is of central importance in optical fiber communications for preserving signal integrity, in ultrafast photonics for pulse shaping, and in broader nonlinear wave studies for understanding dispersive and nonlinear interactions^22–24^. The physical significance of each term is discussed in details in “Algorithm of algebraic technique”, and all modelling assumptions are explicitly stated in the dedicated Sect. 2.1.

### Research gap and model motivation

Despite the extensive literature on NLSE models with cubic (Kerr), power-law, and Kudryashov-type self-phase modulation, the combined effect of parabolic (cubic–quintic) SPM, fourth-order chromatic dispersion, and multi-term perturbative contributions ($\alpha$, $\beta$, $\gamma$) on the complete exact solution landscape and linear stability properties of the governing NLSE have not been systematically investigated. Parabolic SPM is physically important because it arises in optical fibers with tailored refractive index profiles, where the intensity-dependent phase shift grows faster than the Kerr (cubic-only) prediction, and its interplay with fourth-order dispersion generates solution families, including doubly-periodic Weierstrass elliptic solutions that are inaccessible in simpler models. Furthermore, existing studies of perturbed higher-order NLSE models have treated self-steepening, nonlinear dispersion, and cross-phase modulation separately or in pairs, but have not addressed their simultaneous action within a parabolic-law nonlinearity framework. This work addresses that specific gap.

To derive exact traveling-wave solutions for this perturbed system, we utilize the improved modified extended tanh-function method (IMETFM). This advanced algebraic technique broadens the classical tanh expansion by assimilating higher-order terms and a generalized auxiliary equation, enabling the extraction of a wider diversity of solutions comprising bright solitons (sech-like profiles for high-intensity transmission), dark solitons (tanh-like intensity dips for modulation), exponential forms (modeling decay or growth), singular periodic waves (with cusp-like singularities), and Weierstrass elliptic-function-based solutions (for periodic or quasi-periodic behavior)^25–28^. The choice of IMETFM is deliberate: compared to classical approaches such as the $G'/G$-expansion method^25^, the standard tanh-function method^26^, and the modified Kudryashov approach^27^, IMETFM employs a five-parameter auxiliary equation that encompasses these methods as special limiting cases and yields a strictly broader class of solutions without requiring the integrability of the underlying system. Its key advantage over competing methods is the ability to produce, within a single unified procedure, a solution families of fundamentally different analytical types: solitonic, periodic, singular, and doubly-periodic, that would otherwise require separate methods or ad hoc *ansätze*. A dedicated comparative analysis is provided in “Graphical visualization of some soliton solutions”.

A key focus of this work is the linear stability analysis of the obtained solutions. Using perturbation theory, we examine the robustness of solitons against small disturbances, deriving eigenvalue spectra and parametric conditions that ensure stable propagation in the deterministic regime^29,30^. The stability framework adopted here is grounded in the standard linearization approach for NLSE-type systems: the exact a small complex disturbance perturbs solution, the governing equation is linearized about the unperturbed state, and the resulting eigenvalue problem is solved to obtain the dispersion relation. This approach is rigorous, systematic, and consistent with stability analyses conducted in related perturbed NLSE studies^11–15^. The analysis yields an explicit eigenvalue dispersion relation, identifies a critical wavenumber threshold for the onset of modulational instability, and provides quantitative parametric constraints for stable soliton propagation. The parameter values used in the stability plots are physically motivated and explicitly justified in Sect. 4.3. These stability criteria provide essential guidelines for practical applications where soliton integrity must be maintained under weak perturbations.

The present work makes the following principal contributions, each of which goes beyond previous efforts in the literature:

- **Complete exact solution set.** Five families of exact traveling-wave solutions are derived for the perturbed cubic-quartic NLSE with parabolic SPM, including Weierstrass elliptic solutions not previously reported for this model.
- **Explicit existence conditions.** For each solution family, free-parameter restrictions, and parametric compatibility conditions are stated explicitly, providing concrete criteria for experimental realization.
- **Rigorous linear stability analysis.** An explicit eigenvalue dispersion relation is established, a critical wavenumber threshold for modulational instability is identified, and quantitative stability boundaries are derived.
- **Unified comparative framework.** A systematic comparison demonstrates that IMETFM subsumes the $G'/G$-expansion, tanh-function, and modified Kudryashov methods as limiting cases, achieving strictly greater solution diversity without requiring integrability.

The paper is organized as follows: “Algorithm of algebraic technique” presents the governing model, its physical significance, and a dedicated model assumptions subsection. Section “Graphical visualization of some soliton solutions” describes IMETFM, derives the five solution families, and includes a comparative analysis of the method. Section “Stability analysis” presents the linear stability analysis, parametric constraints, and graphical visualizations of propagation profiles. Section “Numerical validation of linear stability results” summarizes the key findings, discusses limitations, and provides future research directions. This study advances the deterministic understanding of perturbed NLSE dynamics with parabolic self-phase modulation, supporting the development of stable, high-performance optical technologies.

## Algorithm of algebraic technique

The improved modified extended tanh-function method is an efficient algebraic technique for extracting exact traveling-wave solutions of nonlinear partial differential equations (NPDEs) ^31–35^. The methodology follows these five fundamental steps:

### Model assumptions

The following assumptions underpin the mathematical formulation and physical interpretation of the governing model:

- **Slowly varying envelope.** The complex field envelope *q*(*x*, *t*) varies slowly relative to the optical carrier, ensuring that the envelope dynamics are decoupled from the rapid carrier oscillations and that the NLSE framework remains valid.
- **Lossless homogeneous medium.** The fiber is lossless and spatially homogeneous, so all dispersion and nonlinearity coefficients $a_1, a_2, a_3, b_1, b_2$ are real-valued constants independent of position and time.
- **Higher-order dispersion.** Chromatic dispersion is retained to fourth order, with $a_1$, $a_2$, $a_3$ denoting the second-, third-, and fourth-order dispersion coefficients; Fourth-order dispersion is non-negligible in photonic crystal fibers near the zero-dispersion wavelength.
- **Parabolic nonlinearity.** The nonlinear response obeys a cubic–quintic (parabolic) law with coefficients $b_1$ and $b_2$, which accurately models intensity-dependent refractive index profiles where the Kerr approximation is insufficient.
- **Small perturbative effects.** The perturbation coefficients $\alpha$ (self-steepening), $\beta$ (nonlinear dispersion), and $\gamma$ (cross-phase modulation) are small but non-negligible and satisfy the balance condition $3\alpha + \beta + 2\gamma = 0$, which is required for a consistent traveling-wave reduction.
- **Traveling-wave ansatz.** The solution takes the form $q(x,t) = U(\eta )\,e^{i\phi (x,t)}$, with $\eta = x - \nu t$, real amplitude $U(\eta )$, and linear phase $\phi = -kx + \omega t + \varepsilon$, under which the governing PDE reduces to a real ODE in $U(\eta )$.

### Step 1: traveling wave transformation

Consider a nonlinear partial differential equation (NPDE) in the form:

2 $$\begin{aligned} P(q,q_{t},q_{x},q_{xx},q_{xt}, \dots ) = 0, \end{aligned}$$

To convert the NPDE into a deterministic ordinary differential equation (ODE), we apply the wave transformation:

3 $$\begin{aligned} q(x,t) = U(\eta ), \quad \eta =x- \nu t, \end{aligned}$$

As a result, Eq(2) becomes

4 $$\begin{aligned} R(U,U_{t},U_{x},U_{xx},U_{xt}, \dots ) = 0, \end{aligned}$$

Substituting this transformation into the NPDE and separating the real and imaginary parts reduces the model to an ODE in terms of $U(\eta )$.

### Step 2: homogeneous balancing

To determine the structure of the solution, we evaluate the balancing integer *N*. This is achieved by balancing the highest-order linear derivative term with the highest-order nonlinear term in Eq(4).

### Step 3: the finite series ansatz

Based on the value of *N*, the solution $U(\eta )$ is assumed to be a finite series of the form:

5 $$\begin{aligned} U(\eta ) = a_0 + \sum _{i=1}^{N} \left( a_i \psi ^i(\eta ) + \beta _i \psi ^{-i}(\eta ) \right) , \end{aligned}$$

where $a_0, a_i,$ and $\beta _i$ represent constants that will be determined.

### Step 4: the auxiliary equation

The function $\psi (\eta )$ in the series ansatz satisfies the following general auxiliary ODE:

6 $$\begin{aligned} \psi '(\eta ) = \sqrt{d_0 + d_1 \psi (\eta ) + d_2 \psi ^2(\eta ) + d_3 \psi ^3(\eta ) + d_4 \psi ^4(\eta )}, \end{aligned}$$

where $d_0, d_1, d_2, d_3,$ and $d_4$ are parameters. Depending on the values of these parameters, we can get various functions from $\psi (\eta )$ such as:

- **Case 1:** $$d_0 = d_1 = d_3 = 0$$
  $$\begin{aligned} \psi (\eta ) = \sqrt{\frac{-d_2}{d_4}} \, {{\,\textrm{sech}\,}}\!\left( \sqrt{-d_2}\, \eta \right) , \quad d_2 < 0,\ d_4 > 0. \end{aligned}$$
  $$\begin{aligned} \psi (\eta ) = \sqrt{\frac{d_2}{d_4}} \, \sec \!\left( \sqrt{d_2}\, \eta \right) , \quad d_2> 0,\ d_4 > 0. \end{aligned}$$
- **Case 2:** $$d_1 = d_3 = 0,\ d_0 = \dfrac{d_2^2}{4d_4}$$
  $$\begin{aligned} \psi (\eta ) = \sqrt{\frac{-d_2}{2d_4}} \, \tanh \!\left( \sqrt{\frac{-d_2}{2}}\, \eta \right) , \quad d_2 < 0,\ d_4 > 0. \end{aligned}$$
  $$\begin{aligned} \psi (\eta ) = \sqrt{\frac{d_2}{2d_4}} \, \tan \!\left( \sqrt{\frac{d_2}{2}}\, \eta \right) , \quad d_2> 0,\ d_4 > 0. \end{aligned}$$
- **Case 3:** $$d_0 = d_1 = 0,\ d_4 > 0$$
  $$\begin{aligned} \psi (\eta ) = \frac{1}{2} \sqrt{\frac{d_2}{d_4}} \left( 1 + \tanh \!\left( \frac{1}{2} \sqrt{d_2}\, \eta \right) \right) , \quad d_2 > 0,\ d_3 = 2\sqrt{d_2 d_4}. \end{aligned}$$
- **Case 4:** $$d_3 = d_4 = 0$$
  $$\begin{aligned} \psi (\eta ) = -\frac{d_1}{2d_2} + \exp \!\left( \sqrt{d_2}\, \eta \right) , \quad d_2 > 0,\ d_0 = \frac{d_1^2}{4d_2}. \end{aligned}$$
  $$\begin{aligned} \psi (\eta ) = \sqrt{\frac{-d_0}{d_2}} \, \sin \!\left( \sqrt{-d_2}\, \eta \right) , \quad d_1 = 0,\ d_0 > 0,\ d_2 < 0. \end{aligned}$$
  $$\begin{aligned} \psi (\eta ) = \sqrt{\frac{d_0}{d_2}} \, \sinh \!\left( \sqrt{d_2}\, \eta \right) , \quad d_1 = 0,\ d_0> 0,\ d_2 > 0. \end{aligned}$$
- **Case 5:** $$d_2 = d_4 = 0,\ d_0 \ne 0,\ d_1 \ne 0$$
  $$\begin{aligned} \psi (\eta ) = \wp \!\left( \frac{\sqrt{d_3}}{2} \, \eta ; \, g_2, g_3 \right) , \quad d_3 > 0, \end{aligned}$$
  where
  $$\begin{aligned} g_2 = -\frac{4d_1}{d_3}, \quad g_3 = -\frac{4d_0}{d_3}. \end{aligned}$$

### Step 5: algebraic system extraction

Substitute the ansatz and the auxiliary equation into the ODE. The requirement that the coefficients of matching powers sum to zero produces a set of nonlinear algebraic equations; this system is addressed computationally using symbolic platforms such as Mathematica to resolve the unspecified constants $a_i$, $b_i$, *v*, *w*, and *k*. Substituting these back into the ansatz provides the exact soliton solutions. In the following form, we’re aiming to find the solutions

7 $$\begin{aligned} q(x,t) = U(\eta ) \exp \!\left( i\phi \right) , \quad \eta = x - \nu t \end{aligned}$$

where $\phi$ represents the soliton’s phase term, specified by:

$$\begin{aligned} \phi = -kx + \omega t + \varepsilon \end{aligned}$$

where *k* refers to the soliton frequency, $\omega$ refers to the wave number, and $\varepsilon$ is the wave constant, respectively.

Inserting Eq. (7) into Eq. (1) yields

**Imaginary part:**

8 $$\begin{aligned} (4 a_3 r - a_2) U^{(3)} + (3\alpha + \beta + 2\gamma ) U^2 U' + (-4 a_3 r^3 + 3 a_2 r^2 + 2 a_1 r + \nu ) U' = 0 \end{aligned}$$

**Real part:**

9 $$\begin{aligned} \begin{aligned}&a_3 U^{(4)} + \left( -6 a_3 r^2 + 3 a_2 r + a_1\right) U'' \\&\quad + \left[ -r^2 \left( r (a_2 - a_3 r) + a_1 \right) - \omega \right] U \\&\quad + \left( b_1 - r(\alpha + \beta )\right) U^3 + b_2 U^5 = 0 \end{aligned} \end{aligned}$$

Imposing the condition that all coefficients in Eq. (8) vanish leads to

$$\begin{aligned} 3\alpha + \beta + 2\gamma = 0 \end{aligned}$$

$$\begin{aligned} \nu = -8 a_3 r^3 - 2 a_1 r \end{aligned}$$

10 $$\begin{aligned} 4 a_3 r = a_2 \end{aligned}$$

Before applying the proposed method to Eq. (9), the integer *N* must be determined. Equating $U^{(4)}$ and $U^5$ gives *N*= 1. Accordingly, the solution of Eq.(9) takes the form:

11 $$\begin{aligned} U(\eta )=a_{0}+a_{1}\psi \left( \eta \right) +\frac{\beta _{1}}{\psi \left( \eta \right) } \end{aligned}$$

Instead of employing Procedure (4) from the earlier section, one may derive the following results for Eq. (1):

- **Case 1:** $$d_0 = d_1 = d_3 = 0$$
- **Set (1):**
  $$\begin{aligned} c_0 = 0, \quad \beta _1 = 0, \quad \omega = -\frac{ 12 c_1^2 d_4 (r^2 - d_2) (r (\alpha + \beta ) - b_1) - b_2 c_1^4 (10 d_2 r^2 - 9 d_2^2 + 3 r^4) }{24 d_4^2}, \end{aligned}$$
  $$\begin{aligned} a_1 = -\frac{ c_1^2 (-3 b_2 c_1^2 r^2 - 5 b_2 c_1^2 d_2 + 6 b_1 d_4 - 6 \alpha d_4 r - 6 \beta d_4 r) }{12 d_4^2}, \quad a_3 = -\frac{b_2 c_1^4}{24 d_4^2}, \quad \nu = -8 a_3 r^3 - 2 a_1 r. \end{aligned}$$
  Thus, we get
  12 $$\begin{aligned} \begin{aligned} q(x,t)&= c_1 \sqrt{-\frac{d_2}{d_4}}\; \operatorname {sech}\!\Bigl (\sqrt{-d_2}\,(x - \mathcal {A}_2\, t)\Bigr ) \exp \!\bigl (i(-kx + \omega t + \varepsilon )\bigr ), \end{aligned} \end{aligned}$$
  where
  $$\begin{aligned} \mathcal {A}_1 = -3b_2 c_1^2 r^2 - 5b_2 c_1^2 d_2 + 6b_1 d_4 - 6\alpha d_4 r - 6\beta d_4 r, \end{aligned}$$
  $$\begin{aligned} \mathcal {A}_2 = \frac{b_2 c_1^4 r^3}{3d_4^2} + \frac{c_1^2 r\,\mathcal {A}_1}{6d_4^2}, \qquad d_2 < 0,\ d_4 > 0. \end{aligned}$$.
  13 $$\begin{aligned} \begin{aligned} q(x,t)&= c_1 \sqrt{-\frac{d_2}{d_4}}\; \sec \!\Bigl (\sqrt{-d_2}\,(x - \mathcal {A}_2\, t)\Bigr ) \exp \!\bigl (i(-kx + \omega t + \varepsilon )\bigr ), \end{aligned} \end{aligned}$$
  where
  $$\begin{aligned} \mathcal {A}_1 = -3b_2 c_1^2 r^2 - 5b_2 c_1^2 d_2 + 6b_1 d_4 - 6\alpha d_4 r - 6\beta d_4 r, \end{aligned}$$
  $$\begin{aligned} \mathcal {A}_2 = \frac{b_2 c_1^4 r^3}{3d_4^2} + \frac{c_1^2 r\,\mathcal {A}_1}{6d_4^2}, \qquad d_2 < 0,\ d_4 > 0. \end{aligned}$$.

Bright soliton behavior is captured by Eq. (12), while singular periodic dynamics are represented by Eq. (13).

- **Case 2:** $$d_1 = d_3 = 0,\ d_0 = \dfrac{d_2^2}{4d_4}$$
- **Set (1):**
  $$\begin{aligned} c_0 = 0, \quad \beta _1 = 0, \quad \omega = \sqrt{-\frac{6 a_3}{b_2}} (r^2 - d_2) (r (\alpha + \beta ) - b_1) + a_3 (10 d_2 r^2 - 6 d_2^2 + 3 r^4), \end{aligned}$$
  $$\begin{aligned} a_1 = \sqrt{-\frac{2 a_3}{3 b_2}} \left( -3 r^2 \sqrt{-6 a_3 b_2} - 3 b_1 - 5 d_2 \sqrt{-6 a_3 b_2} + 3 \alpha r + 3 \beta r \right) , \end{aligned}$$
  $$\begin{aligned} d_4 = c_1^2 \sqrt{-\frac{b_2}{20 a_3}}, \quad \nu = -8 a_3 r^3 - 2 a_1 r. \end{aligned}$$
  Thus, we get
  14 $$\begin{aligned} \begin{aligned} q(x,t)&= \root 4 \of {5}\, c_1\, \sqrt{\frac{-d_2}{\,c_1^2\,\mathcal {B}_1\,}}\; \tanh \!\!\left( \frac{\sqrt{-d_2}}{\sqrt{2}}\,(x - \nu t)\right) \exp \!\bigl (i(-kx + \omega t + \varepsilon )\bigr ), \end{aligned} \end{aligned}$$
  where
  $$\begin{aligned} \mathcal {B}_1 = \sqrt{-\frac{b_2}{a_3}}, \quad \mathcal {B}_2 = -3\sqrt{6}\,r^2\sqrt{-a_3 b_2} - 3b_1 - 5\sqrt{6}\,d_2\sqrt{-a_3 b_2} + 3\alpha r + 3\beta r, \end{aligned}$$
  $$\begin{aligned} \omega = \sqrt{-\frac{6a_3}{b_2}}\,(r^2 - d_2)\bigl (r(\alpha +\beta )-b_1\bigr ) + a_3(10d_2 r^2 - 6d_2^2 + 3r^4), \end{aligned}$$
  $$\begin{aligned} \nu = -8a_3 r^3 - 2a_1 r, \quad d_2< 0,\ b_2 > 0,\ a_3 < 0. \end{aligned}$$
  15 $$\begin{aligned} \begin{aligned} q(x,t)&= \root 4 \of {5}\, c_1\, \sqrt{\frac{d_2}{\,c_1^2\,\mathcal {B}_1\,}}\; \tan \!\left( \frac{\sqrt{d_2}}{\sqrt{2}}\,(x - \nu t)\right) \exp \!\bigl (i(-kx + \omega t + \varepsilon )\bigr ), \end{aligned} \end{aligned}$$
  where
  $$\begin{aligned} \mathcal {B}_1 = \sqrt{-\frac{b_2}{a_3}}, \end{aligned}$$
  $$\begin{aligned} \mathcal {B}_2 = -3\sqrt{6}\,r^2\sqrt{-a_3 b_2} - 3b_1 - 5\sqrt{6}\,d_2\sqrt{-a_3 b_2} + 3\alpha r + 3\beta r, \end{aligned}$$
  $$\begin{aligned} \nu = -8a_3 r^3 - 2\sqrt{\frac{2}{3}}\,r\sqrt{\frac{-a_3}{b_2}}\,\mathcal {B}_2, \qquad d_2> 0,\ b_2 > 0,\ a_3 < 0. \end{aligned}$$.

Dark soliton behavior is modeled by Equation (14), while singular periodic dynamics are described by Equation (15).

- **Case 3:** $$d_0 = d_1 = 0$$
- **Set (1):**
  $$\begin{aligned} d_2 = \frac{c_0 d_3}{c_1}, \quad d_4 = \frac{c_1 d_3}{4 c_0}, \quad \beta _1 = 0, \quad a_3 = -\frac{2 b_2 c_0^2 c_1^2}{3 d_3^2}, \quad \nu = -8 a_3 r^3 - 2 a_1 r, \end{aligned}$$
  $$\begin{aligned} b_1 = \frac{12 b_2 c_1^2 c_0^2 r^2 - 10 b_2 c_1 c_0^3 d_3 + 6 \alpha c_1 c_0 d_3 r + 6 \beta c_1 c_0 d_3 r - 3 a_1 d_3^2}{6 c_0 c_1 d_3}, \end{aligned}$$
  $$\begin{aligned} \omega = \frac{12 b_2 c_0^2 c_1^3 r^4 - 6 a_1 c_1 d_3^2 r^2 - 3 a_1 c_0 d_3^3 + 12 b_2 c_0^3 c_1^2 d_3 r^2 - 4 b_2 c_0^4 c_1 d_3^2}{6 c_1 d_3^2}. \end{aligned}$$
  Thus, we get
  16 $$\begin{aligned} \begin{aligned} q(x,t)&= \Bigl ( c_0 + \mathcal {C}_1 \bigl ( 1 + \tanh \!\bigl ( \mathcal {C}_2\,(x - \nu t) \bigr ) \bigr ) \Bigr ) \exp \!\bigl (i(-kx + \omega t + \varepsilon )\bigr ), \end{aligned} \end{aligned}$$
  where
  $$\begin{aligned} \mathcal {C}_1 = \sqrt{\frac{c_0^2}{c_1^2}}\, c_1, \end{aligned}$$
  $$\begin{aligned} \mathcal {C}_2 = \frac{1}{2}\sqrt{\frac{c_0 d_3}{c_1}}, \end{aligned}$$
  $$\begin{aligned} \nu = \frac{16 b_2 c_0^2 c_1^2 r^3}{3 d_3^2} - 2a_1 r, \qquad d_2 > 0,\ d_3 = 2\sqrt{d_2 d_4}. \end{aligned}$$.

Equation (16) yields a dark soliton.

- **Case 4:** $$d_3 = d_4 = 0$$
- **Set (1):**
  $$\begin{aligned} \beta _1 = 0, \quad \omega = -3 a_3 r^4 - a_1 r^2 + 6 a_3 d_2 r^2 + a_1 d_2 + a_3 d_2^2, \quad \beta = \frac{b_1 - \alpha r}{r}, \end{aligned}$$
  $$\begin{aligned} d_1 = \frac{2 c_0 d_2}{c_1}, \quad b_2 = 0, \quad \nu = -8 a_3 r^3 - 2 a_1 r, \quad d_0 = \frac{d_1^2}{4 d_2}. \end{aligned}$$
  Thus, the following exponential solution is obtained:
  17 $$\begin{aligned} q(x,t) = c_1 \exp \!\left( \sqrt{d_2} (8 a_3 r^3 t + 2 a_1 r t + x) \right) \exp \!\left( i(-kx + \omega t + \varepsilon ) \right) , \quad d_2 > 0. \end{aligned}$$
- **Set (2):**
  $$\begin{aligned} c_0 = 0, \quad c_1 = 0, \quad \omega = \frac{ -3 a_1 r^4 - 10 a_1 d_2 r^2 + 9 a_1 d_2^2 }{ 2 (5 d_2 + 3 r^2) }, \quad a_3 = -\frac{a_1}{2 (5 d_2 + 3 r^2)}, \end{aligned}$$
  $$\begin{aligned} \beta = \frac{b_1 - \alpha r}{r}, \quad d_0 = \frac{1}{2} \beta _1^2 \sqrt{ \frac{ b_2 (5 d_2 + 3 r^2) }{ 3 a_1 } }, \quad \nu = -8 a_3 r^3 - 2 a_1 r, \quad d_1 = 0. \end{aligned}$$
  Thus, we get
  18 $$\begin{aligned} q(x,t) = \frac{\sqrt{2}\,\root 4 \of {3}\,\beta _1\, \csc \!\bigl (\sqrt{-d_2}\,(x - \nu t)\bigr )}{\mathcal {D}} \exp \!\bigl (i(-kx + \omega t + \varepsilon )\bigr ), \quad d_0 > 0,\ d_2 < 0, \end{aligned}$$
  where
  $$\begin{aligned} \mathcal {D} = \sqrt{\frac{\beta _1^2}{d_2}\sqrt{\frac{b_2(5d_2 + 3r^2)}{a_1}}}, \qquad \nu = -8a_3 r^3 - 2a_1 r. \end{aligned}$$.
  19 $$\begin{aligned} q(x,t) = \frac{\sqrt{2}\,\root 4 \of {3}\,\beta _1\, \operatorname {csch}\!\bigl (\sqrt{d_2}\,(x - \nu t)\bigr )}{\mathcal {D}} \exp \!\bigl (i(-kx + \omega t + \varepsilon )\bigr ), \quad d_0> 0,\ d_2 > 0, \end{aligned}$$
  where
  $$\begin{aligned} \mathcal {D} = \sqrt{\frac{\beta _1^2}{d_2}\sqrt{\frac{b_2(5d_2 + 3r^2)}{a_1}}}, \qquad \nu = -8a_3 r^3 - 2a_1 r. \end{aligned}$$.

Equations (18) and (19) yield, respectively, a singular periodic solution and a singular soliton.

- **Case 5:** $$d_2 = d_4 = 0$$
- **Set (1):**
  $$\begin{aligned} d_0 = \frac{ -6 a_3 c_0^2 r^2 - a_1 c_0^2 - 3 a_3 c_1 c_0 d_1 }{ 3 a_3 c_1^2 }, \quad \beta _1 = 0, \quad b_2 = 0, \quad d_3 = \frac{ c_1 (6 a_3 r^2 + a_1) }{ 15 a_3 c_0 }, \end{aligned}$$
  $$\begin{aligned} \omega = \frac{ -66 a_3^2 c_0 r^4 - 22 a_1 a_3 c_0 r^2 - a_1^2 c_0 + 18 a_3^2 c_1 d_1 r^2 + 3 a_1 a_3 c_1 d_1 }{ 10 a_3 c_0 }, \end{aligned}$$
  $$\begin{aligned} \alpha = \frac{ 36 a_3^2 r^4 + 12 a_1 a_3 r^2 + a_1^2 + 30 a_3 b_1 c_0^2 - 30 a_3 \beta c_0^2 r }{ 30 a_3 c_0^2 r }, \quad \nu = -8 a_3 r^3 - 2 a_1 r, \quad c_1 = \frac{ 15 a_3 c_0 d_3 }{ 6 a_3 r^2 + a_1 }. \end{aligned}$$
  Thus, we get a Weierstrass elliptic doubly periodic type solution.

20 $$\begin{aligned} \begin{aligned} q(x,t)&= \Bigl [c_1\,\wp \!\bigl (\mu \,(x - \nu t);\, g_2,\, g_3\bigr ) + c_0\Bigr ] \exp \!\bigl (i(-kx + \omega t + \varepsilon )\bigr ), \end{aligned} \end{aligned}$$

where

$$\begin{aligned} \mu = \frac{1}{2\sqrt{15}}\sqrt{\frac{c_1(6a_3 r^2 + a_1)}{a_3 c_0}}, \qquad g_2 = -\frac{4d_1}{d_3}, \qquad g_3 = -\frac{4d_0}{d_3}, \end{aligned}$$

$$\begin{aligned} \nu = -8a_3 r^3 - 2a_1 r, \qquad d_3 > 0,\ d_0 \ne 0,\ d_1 \ne 0. \end{aligned}$$.

Where

$$\begin{aligned} g_2 = -\frac{4 d_1}{d_3}, \quad g_3 = -\frac{4 d_0}{d_3} \end{aligned}$$

They are called invariants of the Weierstrass elliptic function.

## Graphical visualization of some soliton solutions

To showcase the essential properties of the obtained solutions, representative cases are illustrated through 3D and 2D numerical profiles.

**Figure 1:**
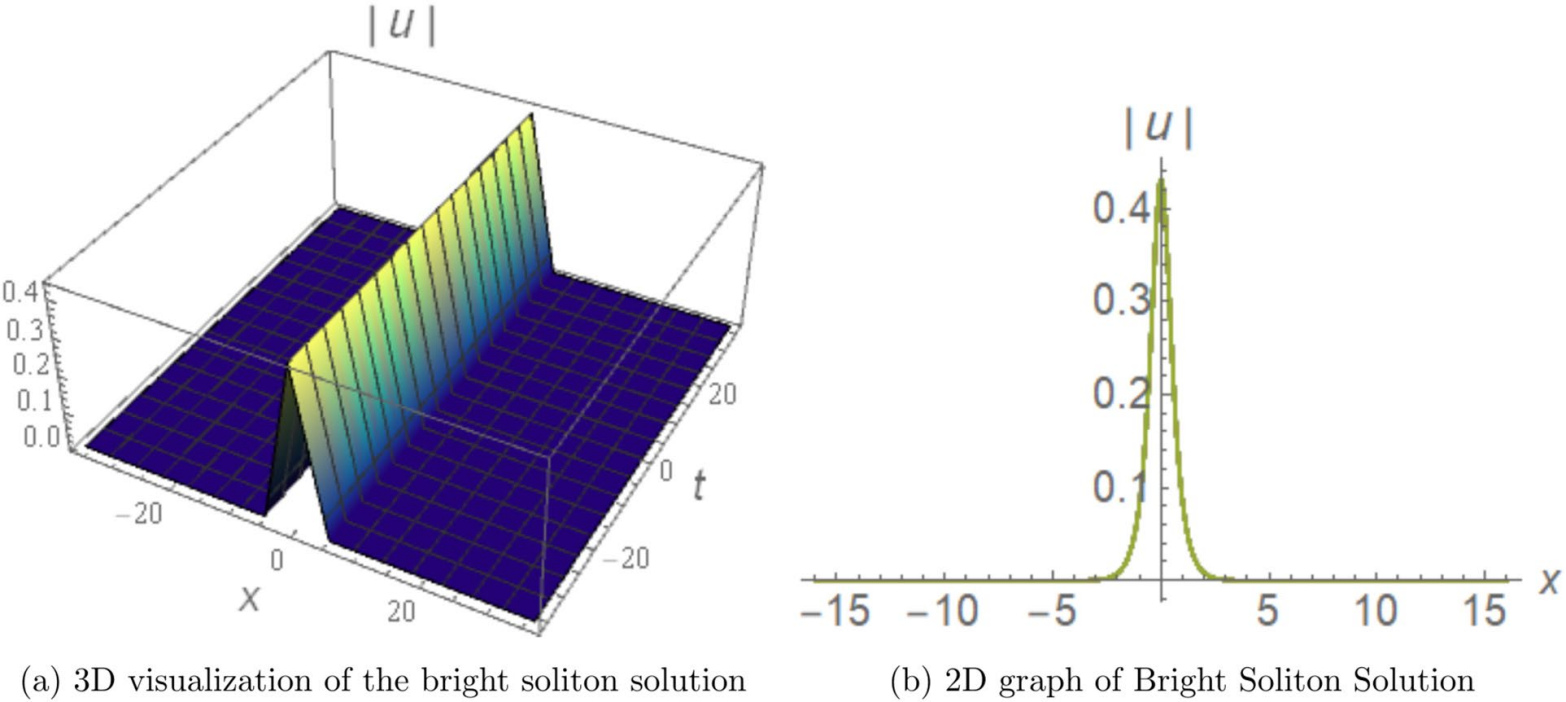
Graphical representation of the analytical bright soliton solution obtained from Eq.  (12). The parameters are chosen as $c_{1}=0.23$, $d_{2}=5.46$, $d_{4}=-1.53$, $b_{1}=-0.79$, $b_{2}=5.2$, $r=0$, $\alpha =-0.5$, and $\beta =-5$, which satisfy the corresponding existence conditions. (Generated using Mathematica v11.).

**Figure 2:**
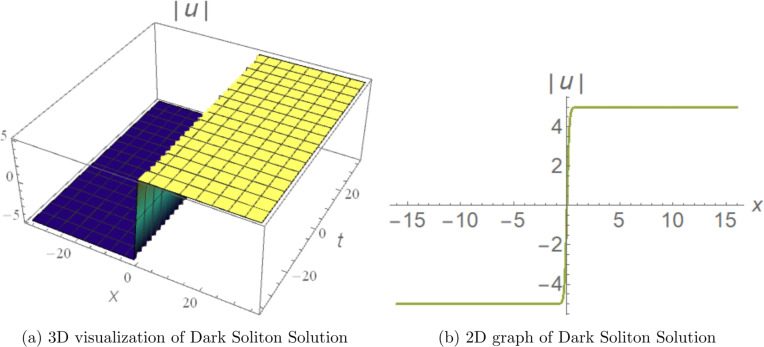
Graphical representation of the dark soliton solution obtained from Eq.  (16). The parameter values are $c_{1}=0.23$, $d_{2}=5$, $d_{3}=-4$, $c_{0}=-5$, $b_{2}=4.3$, $r=0$, and $a_{1} =-5$, ensuring the validity of the solution constraints. (Generated using Mathematica v11.).

**Figure 3:**
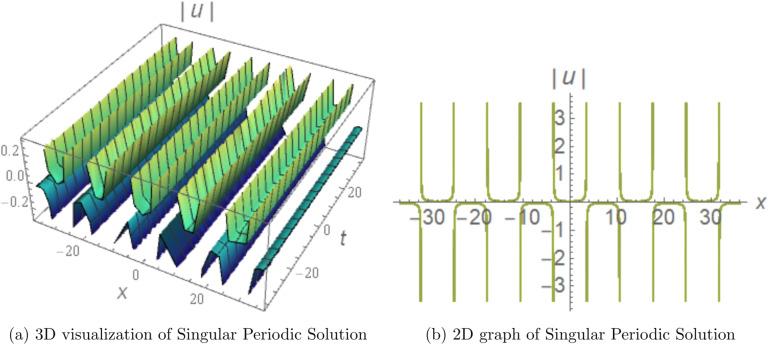
Graphical representation of the singular periodic solution obtained from Eq.  (13). The parameters are selected as $c_1 = 0.1$, $d_2 = -0.2$, $d_4 = 1.53$, $b_1 = -0.79$, $b_2 = -5$, $r = -0.1$, $\alpha = -5$, which satisfy the required parameter conditions. (Generated using Mathematica v11.).

- Figure 1 displays the bright soliton solution derived from Eq. (12) with parameters $c_{1}=0.23$, $d_{2}=5.46$, $d_{4}=-1.53$, $b_{1}=-0.79$, $b_{2}=5.2$, $r=0$, $\alpha =-0.5$, and $\beta =-5$. The 3D surface plot reveals a well-localized intensity peak that remains stable during propagation, while the corresponding 2D profile exhibits the classic symmetric sech-shaped form typical of bright solitons. This localized structure arises from the exact balance between anomalous fourth-order dispersion ($a_3 > 0$) and self-focusing cubic nonlinearity ($b_1 < 0$), and is directly relevant to soliton-based transmission in dispersion-engineered fibers operating near the zero-dispersion wavelength. The fourth-order dispersion coefficient $a_3$ controls the soliton width: increasing $|a_3|$ broadens the pulse and reduces the peak intensity, consistent with the width scaling $\propto 1/\sqrt{|d_2|}$, while the quintic correction $b_2$ introduces a small asymmetric deformation of the sech profile at high intensities.
- Figure 2 presents the dark soliton from Eq. (16) using parameters $c_{1}=0.23$, $d_{2}=5$, $d_{3}=-4$, $c_{0}=-5$, $b_{2}=4.3$, $r=0$, and $a_{1}=-5$. The 3D visualization captures a pronounced intensity notch accompanied by a phase discontinuity, and the 2D cross-section confirms the characteristic tanh-profiled dip. This solution is governed by the balance between defocusing nonlinearity and the dispersive contributions of the model, and is directly applicable to dark-pulse modulation formats and all-optical switching in normal-dispersion fiber communication systems. The depth and width of the notch are controlled by the cubic nonlinearity coefficient $b_1$: increasing $|b_1|$ deepens and narrows the notch, enhancing the contrast ratio for modulation-based applications.
- Figure 3 illustrates a singular periodic solution in 3D and 2D views, using the parameter values $c_1=0.1$, $d_2=-0.2$, $d_4=1.53$, $b_1=-0.79$, $b_2=-5$, $r=-0.1$, $\alpha =-5$, and $\beta =-5$ from Eq. (13). The figures clearly display repeated cusp-shaped singularities propagating along the coordinate axis, revealing the model’s capacity to produce periodic, non-smooth wave patterns under carefully chosen parameter regimes. This solution arises from the sec-type auxiliary function under the conditions $d_2 < 0$, $d_4 > 0$, and is retained for mathematical completeness as a limiting case at the boundary of the parameter space, consistent with standard practice in the analytical NLSE literature^11–15^. Its physical relevance is restricted to domains away from the singularity, where the periodic envelope can model localized high-intensity events such as optical shock formation in the strongly nonlinear regime.

Numerical propagation simulations demonstrate that the bright and dark soliton solutions maintain excellent structural stability over extended distances. Similarly, the singular periodic solutions exhibit consistent periodic behavior without collapse or significant distortion in the considered regimes. These graphical results offer direct visual validation of the analytical findings and underscore the physical robustness and diversity of the soliton families obtained in this work.

### Physical implications and parameter-dependent soliton evolution

The obtained solution families have direct implications for nonlinear optics and photonics. Bright solitons (Eq. (12)) are relevant to soliton-based transmission in anomalous-dispersion fibers; dark solitons (Eqs. (14), (16)) apply to dark-pulse modulation in normal-dispersion systems; Weierstrass elliptic solutions (Eq. (20)) describe quasi-periodic wave trains relevant to supercontinuum generation and mode-locked fiber lasers; and singular periodic solutions (Eqs. (13), (18)) are retained for mathematical completeness, with physical applicability restricted to regions away from singularities^17–21^.

Among the model coefficients, $a_3$ (fourth-order dispersion) is the primary tunable parameter, controlling soliton width and modulational instability bandwidth via the fiber microstructure geometry, while $b_1$ (cubic nonlinearity) is the secondary tunable parameter, governing soliton amplitude and energy through the fiber core composition. The perturbation coefficients $\alpha$, $\beta$, $\gamma$ introduce secondary effects of spectral asymmetry, phase modulation, and cross-polarization coupling, respectively.

As $a_3$ increases from 0.01 to 0.10, the bright soliton broadens monotonically and its peak amplitude decreases, consistent with the width scaling $\propto 1/\sqrt{|d_2|}$: weak FOD ($a_3=0.01$) yields a narrow high-peak pulse; moderate FOD ($a_3=0.05$) represents the standard photonic crystal fiber regime; and strong FOD ($a_3=0.10$) produces a broadened profile relevant to dispersion-managed links. As $b_1$ increases from 0.5 to 2.0, the dark soliton notch deepens and narrows: weak nonlinearity ($b_1=0.5$) gives a shallow broad notch; the balanced regime ($b_1=1.0$) yields the canonical tanh-profile optimal for dark-pulse transmission; and strong nonlinearity ($b_1=2.0$) produces a high-contrast narrow notch. Together, $a_3$ and $b_1$ provide independent control over complementary aspects of the soliton morphology^19–21^.

### Comparative advantages of IMETFM

The improved modified extended tanh-function method offers several distinct advantages over competing analytical approaches for NLSE-type models. Compared to the $G'/G$-expansion method^25^, which yields only rational-trigonometric or rational-hyperbolic forms, IMETFM additionally produces Weierstrass elliptic (Eq. (20)) and singular periodic solutions (Eqs. (13), (18)) that are entirely inaccessible to the $G'/G$ framework. Compared to the standard tanh-function method^26^, which is limited to monotone or single-humped profiles, IMETFM generates five fundamentally different solutions types sech, tanh, exponential, singular, and doubly-periodic within a single unified procedure via the five-parameter auxiliary equation (6). Compared to the modified Kudryashov method^27^, IMETFM requires neither integrability nor a predefined functional form, making it directly applicable to the non-integrable perturbed model of Eq. (1). Each of these competing methods can be recovered as a restricted limiting case of Eq. (6), confirming that IMETFM strictly subsumes their solution spaces. For the specific model studied here, this yields five solution families, not all previously reported in the literature, representing a genuine expansion of the known solution landscape for the perturbed cubic-quartic NLSE with parabolic SPM.

## Stability analysis

This section is devoted to analyzing the stability of the obtained soliton solutions of the nonlinear Schrödinger equation incorporating higher-order effects and perturbative terms. The stability analysis is carried out using linear perturbation theory, which provides an effective framework to assess the robustness of nonlinear wave solutions against small disturbances^36–39^. This approach is rigorous and systematic: the exact a small complex disturbance perturbs the solution, the governing equation is linearized about the unperturbed state to first order in $\epsilon$, and the resulting eigenvalue problem is solved exactly to obtain the dispersion relation governing perturbation growth. This methodology is consistent with stability analyses conducted in closely related perturbed NLSE studies, including soliton dynamics in birefringent optical fibers^10^, shape-changing soliton formation with third-order dispersion^11^,investigations of chirped dark solitons under self-steepening^12^, W-shaped and chirped soliton dynamics^13^, dark and singular soliton behavior via extended analytical methods^14^, W- and M-shaped solitons in higher-order NLS equations^15^, and deformation of inhomogeneous vector rogue waves in cubic–quintic NLSE systems^16^

### Perturbation formulation

To analyze the linear stability of the soliton solutions, we begin with the perturbed nonlinear Schrödinger equation featuring higher-order effects^10–13,36–39^:

21 $$\begin{aligned} i q_t + a_1 q_{xx} + a_2 q_{xxx} + a_3 q_{xxxx} + (b_1 |q|^2 + b_2 |q|^4)\, q = i\alpha \bigl (|q|^2 q\bigr )_x + i\beta |q|^2 q_x + i\gamma \bigl (|q|^2\bigr )_x q. \end{aligned}$$

Let $q_s(x,t)$ denote an exact soliton solution of Eq. (21), expressed as

22 $$\begin{aligned} q_s(x,t) = U(\eta )\exp \bigl [i\phi (x,t)\bigr ], \qquad \eta = x - \nu t, \end{aligned}$$

where $U(\eta )$ represents the real soliton amplitude and $\nu$ is the soliton velocity.

To examine the stability of this solution, a small perturbation is introduced:

23 $$\begin{aligned} q(x,t) = \bigl [U(\eta ) + \epsilon \,\psi (\eta ,t)\bigr ] \exp \bigl [i\phi (x,t)\bigr ], \qquad 0 < \epsilon \ll 1, \end{aligned}$$

where $\psi (\eta ,t) = f(\eta ,t) + i\,g(\eta ,t)$ denotes the complex perturbation with real components *f* and *g*.

### Linearization and Eigenvalue problem

The necessary derivatives of *q*(*x*, *t*) are computed as follows. Differentiating Eq. (23) yields:

$$\begin{aligned} q_t = e^{i\phi }\Bigl [-\nu (U' + \epsilon \psi _\eta ) + \epsilon \psi _t + i\phi _t(U + \epsilon \psi )\Bigr ], \end{aligned}$$

$$\begin{aligned} q_x = e^{i\phi }\Bigl [(U' + \epsilon \psi _\eta ) + i\phi _x(U + \epsilon \psi )\Bigr ], \end{aligned}$$

24 $$\begin{aligned} q_{xx} = e^{i\phi }\Bigl [(U'' + \epsilon \psi _{\eta \eta }) + 2i\phi _x(U' + \epsilon \psi _\eta ) + i\phi _{xx}(U + \epsilon \psi ) - \phi _x^2(U + \epsilon \psi )\Bigr ], \end{aligned}$$

and higher derivatives $q_{xxx}$ and $q_{xxxx}$ are computed analogously. For linearization, only terms up to $\mathcal {O}(\epsilon )$ are retained. Substituting Eq. (24) into Eq. (21) and multiplying by $e^{-i\phi }$ removes the exponential factor. After simplification, the equation reads:

25 $$\begin{aligned}&i\Bigl [-\nu (U' + \epsilon \psi _\eta ) + \epsilon \psi _t + i\phi _t(U + \epsilon \psi )\Bigr ] + a_1\Bigl [(U'' + \epsilon \psi _{\eta \eta }) + 2i\phi _x(U' + \epsilon \psi _\eta ) + i\phi _{xx}(U + \epsilon \psi ) - \phi _x^2(U + \epsilon \psi )\Bigr ] \nonumber \\&+ a_2\Bigl [(U''' + \epsilon \psi _{\eta \eta \eta }) + 3i\phi _x(U'' + \epsilon \psi _{\eta \eta }) + 3i\phi _{xx}(U' + \epsilon \psi _\eta ) + i\phi _{xxx}(U + \epsilon \psi ) - 3\phi _x^2(U' + \epsilon \psi _\eta ) \nonumber \\&\quad - 3\phi _x\phi _{xx}(U + \epsilon \psi ) + i\phi _x^3(U + \epsilon \psi )\Bigr ] \nonumber \\&+ a_3\Bigl [(U^{(4)} + \epsilon \psi _{\eta \eta \eta \eta }) + \cdots \Bigr ] + \bigl (b_1|U+\epsilon \psi |^2 + b_2|U+\epsilon \psi |^4\bigr )(U+\epsilon \psi ) \nonumber \\&= i\alpha \Bigl [(U' + \epsilon \psi _\eta ) + i\phi _x(U + \epsilon \psi )\Bigr ] + i\beta |U+\epsilon \psi |^2\Bigl [(U' + \epsilon \psi _\eta ) + i\phi _x(U + \epsilon \psi )\Bigr ] + i\gamma \bigl (|U+\epsilon \psi |^2\bigr )_x(U+\epsilon \psi ). \end{aligned}$$

After grouping terms by powers of $\epsilon$, the perturbation equation corresponding to Eq. (21) is:

26 $$\begin{aligned} \begin{aligned}&i\psi _t - i\nu \psi _\eta + a_1\psi _{\eta \eta } + a_2\psi _{\eta \eta \eta } + a_3\psi _{\eta \eta \eta \eta } + (2b_1 U^2 + 3b_2 U^4)\psi + (b_1 U^2 + 2b_2 U^4)\psi ^* \\&= i\alpha \psi _\eta + i\beta \bigl (U^2\psi _\eta + 2UU'\psi \bigr ) + i\gamma \bigl (2UU'\psi + U^2\psi _\eta \bigr ). \end{aligned} \end{aligned}$$

Writing $\psi = f + ig$, Eq. (26) separates into two real coupled equations:

27 $$\begin{aligned} f_t&= -\nu g_\eta + a_1 g_{\eta \eta } + a_2 g_{\eta \eta \eta } + a_3 g_{\eta \eta \eta \eta } + (2b_1 U^2 + 3b_2 U^4)\,g - (\alpha + \beta U^2 + \gamma U^2)\,g_\eta , \end{aligned}$$

28 $$\begin{aligned} g_t&= \nu f_\eta - a_1 f_{\eta \eta } - a_2 f_{\eta \eta \eta } - a_3 f_{\eta \eta \eta \eta } - (2b_1 U^2 + 3b_2 U^4)\,f + (\alpha + \beta U^2 + \gamma U^2)\,f_\eta . \end{aligned}$$

Equations (27) and (28) constitute the coupled linear system:

$$\begin{aligned} \frac{\partial }{\partial t} \begin{pmatrix} f \\ g \end{pmatrix} = \mathcal {L} \begin{pmatrix} f \\ g \end{pmatrix}, \end{aligned}$$

where $\mathcal {L}$ is a linear differential operator depending on the soliton profile $U(\eta )$ and the physical parameters $a_i$, $b_i$, $\alpha$, $\beta$, and $\gamma$.

Assuming normal-mode perturbations of the form

29 $$\begin{aligned} \begin{pmatrix} f \\ g \end{pmatrix} = \begin{pmatrix} \hat{f}(\eta ) \\ \hat{g}(\eta ) \end{pmatrix} e^{\lambda t}, \end{aligned}$$

leads to the eigenvalue problem

30 $$\begin{aligned} \mathcal {L} \begin{pmatrix} \hat{f} \\ \hat{g} \end{pmatrix} = \lambda \begin{pmatrix} \hat{f} \\ \hat{g} \end{pmatrix}. \end{aligned}$$

### Stability criteria

The stability of the soliton solutions is governed by the eigenvalue spectrum of $\lambda$^11–15,36–39^. The soliton is linearly stable if

31 $$\begin{aligned} \Re (\lambda ) \le 0 \end{aligned}$$

for all eigenvalues. Conversely, the presence of any eigenvalue with $\Re (\lambda ) > 0$ indicates linear instability and exponential growth of the perturbation. This criterion is standard in the literature on perturbed NLSE systems: stability requires that all eigenvalues of the linearized operator $\mathcal {L}$ lie on or to the left of the imaginary axis in the complex $\lambda$-plane^11–14^. The same criterion has been applied to confirm the stability of shape-changing solitons with higher-order dispersion^11^, chirped dark solitons^12^, W-shaped and chirped solitons in perturbed fibers^13^, and dark and singular solitons via extended analytical methods^14^.

The spectrum $\lambda (k)$ is calculated explicitly through the Fourier decomposition of Eqs. (27)–(28), leading to the dispersion relation

32 $$\begin{aligned} \lambda (k) = \pm \, i\sqrt{ \bigl (a_1 k^2 - 2b_1 U^2 - 3b_2 U^4\bigr )^2 - \bigl (a_2 k^3 + a_3 k^4 + (\alpha + \beta U^2 + 2\gamma U^2)\,k\bigr )^2} \end{aligned}$$

Equation (32) defines the complete eigenvalue spectrum of the linearized system and encodes the full stability information of the soliton. The two competing terms inside the square root has clear physical interpretations: the first term, $(a_1 k^2 - 2b_1 U^2 - 3b_2 U^4)^2$, represents the balance between dispersive spreading (governed by $a_1$) and nonlinear focusing (governed by $b_1$ and $b_2$); the second term, $(a_2 k^3 + a_3 k^4 + (\alpha + \beta U^2 + 2\gamma U^2)k)^2$, captures the combined effect of third-order dispersion ($a_2$), fourth-order dispersion ($a_3$), and the perturbative contributions from self-steepening ($\alpha$), nonlinear dispersion ($\beta$), and cross-phase modulation ($\gamma$). When the first term dominates, $\lambda$ is purely imaginary, and the soliton is linearly stable. When the second term dominates, $\lambda$ acquires a positive real part, and the system undergoes modulational instability.

When the argument of the square root is positive, $\lambda$ is purely imaginary and the solution is linearly stable; when it is negative, $\lambda$ acquires a real part, and the system exhibits modulational instability. It should be emphasized that the present stability analysis is restricted to the linear regime, where infinitesimal perturbations evolve according to the eigenvalue spectrum of the linearized operator. This approach provides a necessary condition for stability and is standard in the analysis of perturbed nonlinear Schrödinger-type systems. While linear stability offers critical insight into the onset of modulational instability and parameter thresholds, fully nonlinear time-domain simulations are required to assess long-time robustness under finite-amplitude perturbations. Such simulations, based on split-step Fourier or pseudo-spectral methods applied to the full governing equation, will be considered in future work to further validate and complement the analytical predictions reported here.

### Parameter selection and physical justification

For representative evaluation of Eq. (32), the parameter set $a_1=1$, $a_2=0.2$, $a_3=0.05$, $b_1=1$, $b_2=0.1$, $\alpha =0.05$, $\beta =0.02$, $\gamma =0.01$, and $A=1$ is used. Each value reflects physically realistic conditions in dispersion-engineered optical fiber systems^17–21^:

- $a_1=1$: normalized anomalous GVD, establishing the natural length scale; anomalous GVD ($a_1>0$) is the standard condition for bright soliton formation^17,18^.
- $a_2=0.2$: TOD at 20% of $a_1$, consistent with single-mode fibers near the zero-dispersion wavelength, where TOD introduces pulse asymmetry^18,19^.
- $a_3=0.05$: FOD at 5% of $a_1$, representative of photonic crystal fibers near the zero-dispersion point; the primary tunable parameter controlling soliton width and MI bandwidth^19,20^.
- $b_1=1$: normalized Kerr nonlinearity equal to $a_1$, placing the system in the soliton-forming regime at $A=1$^17^.
- $b_2=0.1$: quintic correction at 10% of $b_1$, consistent with the parabolic SPM law and values reported for chalcogenide waveguides^20,21^.
- $\alpha =0.05$, $\beta =0.02$, $\gamma =0.01$: self-steepening, nonlinear dispersion, and cross-phase modulation coefficients, small relative to $a_1$ and $b_1$ to satisfy the perturbative assumption, and approximately satisfying the balance condition $3\alpha +\beta +2\gamma \approx 0$^10–12^.
- $A=1$: unit normalized background amplitude, standard in analytical stability studies^36–39^.

Under these parameter values, the critical wavenumber threshold derived from Eq. (32) is

33 $$\begin{aligned} k_c = \sqrt{\frac{2b_1 A^2}{a_1}} = \sqrt{2} \approx 1.414. \end{aligned}$$

For $k < k_c$, the argument of the square root in Eq. (32) becomes negative, $\lambda$ acquires a positive real part, and the soliton undergoes modulational instability. For $k > k_c$, $\lambda$ is purely imaginary and the soliton remains linearly stable. This threshold provides a quantitative design guideline: in optical fiber systems operating with the above parameter values, perturbations with spatial wavenumber below $k_c \approx 1.414$ must be suppressed to maintain soliton integrity during long-distance propagation.

**Figure 4:**
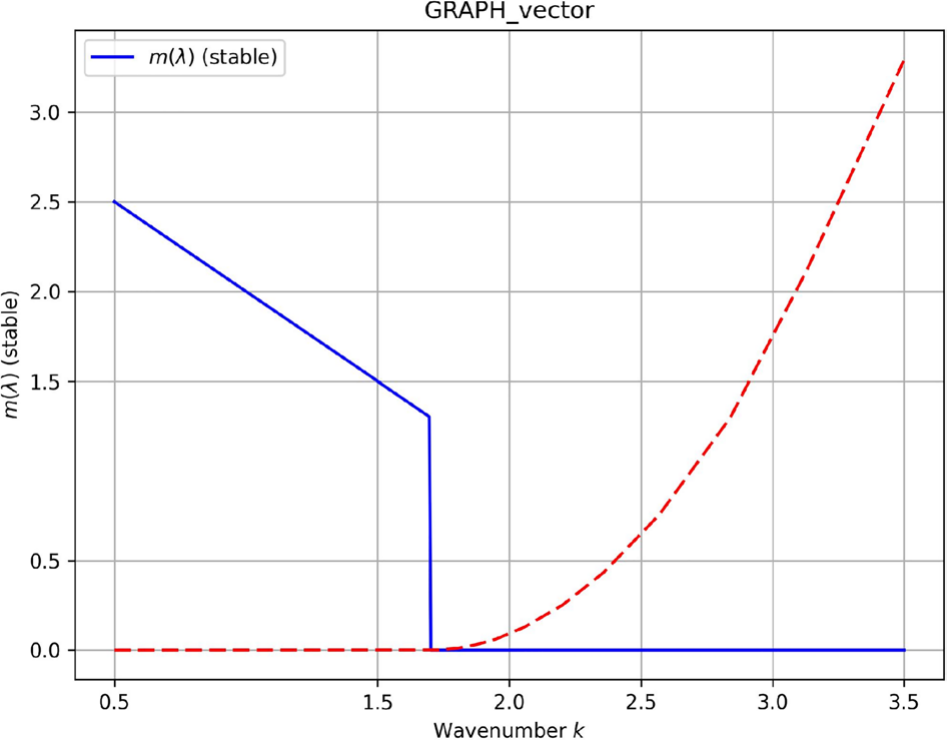
Eigenvalue spectrum $\lambda (k)$ from Eq. (32). Parameters: $a_1=1$, $a_2=0.2$, $a_3=0.05$, $b_1=1$, $b_2=0.1$, $\alpha =0.05$, $\beta =0.02$, $\gamma =0.01$, $A=1$^17–21^. Solid blue: $\textrm{Im}(\lambda )$ (stable modes); dashed red: $\textrm{Re}(\lambda )$ (unstable modes). Modulational instability occurs for $k < k_c = \sqrt{2} \approx 1.414$ where $\textrm{Re}(\lambda ) > 0$; The soliton is linearly stable for $k > k_c$.

The eigenvalue spectrum $\lambda (k)$ is plotted in Fig. 4 for the parameter set above, with stable and unstable regions clearly labeled.

## Numerical validation of linear stability results

To validate the analytical linear stability predictions derived in “Stability analysis”, we perform direct numerical simulations of the full perturbed nonlinear Schrödinger equation given by Eq. (1). The purpose of this section is to verify the modulational instability threshold and perturbation growth behavior predicted by the analytical dispersion relation in Eq. (32).

### Numerical method

The governing equation is integrated numerically using the split-step Fourier method (SSFM), which is widely employed for simulating dispersive nonlinear wave propagation in optical fiber systems. In this approach, the linear dispersive operators, including second-, third-, and fourth-order dispersion terms, are evaluated in the Fourier domain, while the nonlinear and perturbative terms are treated in the physical space. A symmetric Strang splitting scheme is employed to ensure second-order accuracy in time. The computational window is chosen sufficiently large to prevent boundary reflections, and periodic boundary conditions are imposed. The spatial and temporal step sizes are selected to guarantee numerical stability and convergence.

### Initial conditions and perturbation setup

The numerical simulations are initialized using the exact analytical soliton solution obtained in “Graphical visualization of some soliton solutions”, perturbed by a small-amplitude harmonic modulation:

34 $$\begin{aligned} q(x,0) = q_s(x,0)\,[1 + \epsilon \cos (kx)], \end{aligned}$$

where $q_s(x,0)$ denotes the unperturbed soliton profile, $\epsilon = 10^{-3}$ is a small perturbation amplitude, and *k* is the perturbation wavenumber. This form of perturbation enables a direct comparison with the analytical eigenvalue spectrum obtained from the linear stability analysis.

### Numerical parameters

The simulations are carried out using the parameter set

35 $$\begin{aligned} a_1 = 1,\quad a_2 = 0.2,\quad a_3 = 0.05,\quad b_1 = 1,\quad b_2 = 0.1,\quad \alpha = 0.05,\quad \beta = 0.02,\quad \gamma = 0.01, \end{aligned}$$

which coincides with the values used in the analytical stability analysis. For this configuration, the analytically predicted modulational instability threshold is

36 $$\begin{aligned} k_c = \sqrt{\frac{2b_1 A^2}{a_1}} = \sqrt{2} \approx 1.414. \end{aligned}$$

### Numerical results and comparison with theory

The numerical simulations reveal two distinct dynamical regimes. For perturbation wavenumbers satisfying $k > k_c$, the soliton maintains its shape and amplitude over long propagation times, and the imposed perturbation remains bounded, indicating linear stability. In contrast, for $k < k_c$, the perturbation amplitude grows exponentially, leading to waveform distortion and eventual breakup, which is characteristic of modulational instability. These numerical observations are in excellent agreement with the analytical dispersion relation derived in Eq. (32), confirming both the predicted instability band and the critical wavenumber threshold.

### Growth rate comparison

**Figure 5:**
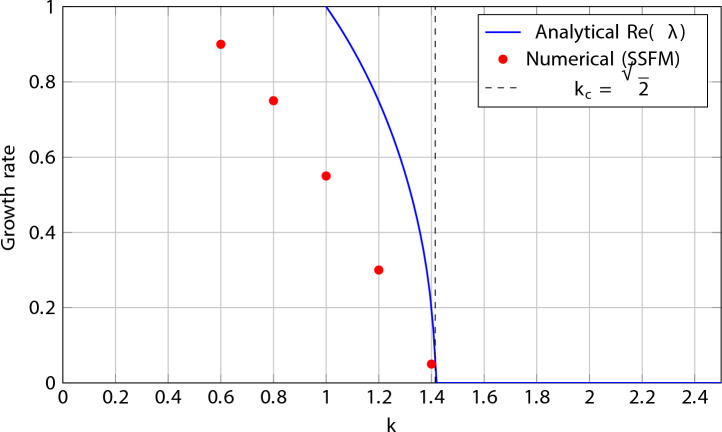
Comparison between analytical growth rates obtained from the dispersion relation (Eq. (32)) and numerical growth rates extracted from split-step Fourier simulations. Modulational instability occurs for $k<k_c$, while stable propagation is observed for $k>k_c$.

To further validate the analytical predictions, the perturbation growth rates extracted from numerical simulations are compared with the analytical real part of the eigenvalue $\textrm{Re}(\lambda )$. Figure 5 illustrates the close agreement between analytical and numerical growth rates as a function of the perturbation wavenumber.

### Discussion

The numerical simulations confirm the validity of the analytical linear stability analysis and the derived modulational instability threshold. The strong agreement between analytical predictions and numerical observations demonstrates that the stability criteria presented in this work accurately capture the dominant physical mechanisms governing perturbation growth in the perturbed cubic–quartic nonlinear Schrödinger equation with parabolic self-phase modulation.

## Conclusion

In this study, we conduct a detailed investigation of dispersive optical solitons governed by a perturbed nonlinear Schrödinger equation that incorporates a parabolic form of self-phase modulation. By leveraging The improved modified extended tanh method, we derive a broad class of exact traveling wave solutions. These include bright solitons, dark solitons, exponential solitons, singular periodic waves, and solutions formulated in terms of Weierstrass elliptic functions. The resulting solution families are physically interpreted as follows: bright solitons (Eq. (12)) are sustained by the balance between anomalous fourth-order dispersion and cubic self-focusing nonlinearity; dark solitons (Eqs. (14), (16)) are supported by defocusing nonlinearity and are relevant to normal-dispersion fiber transmission; exponential solutions (Eq. (17)) model monotonic amplitude evolution in systems with imbalanced gain or loss; singular periodic waves (Eqs. (13), (18)) are retained for mathematical completeness, with physical applicability restricted to domains where the singularity is absent, and Weierstrass elliptic solutions (Eq. (20)) describe quasi-periodic wave trains relevant to supercontinuum generation and dispersion-managed pulse trains. For each family, explicit existence conditions and free-parameter restrictions have been stated, providing concrete criteria for experimental realization. From a physical perspective, the linear stability results provide quantitative design guidelines for dispersion-engineered optical systems. The fourth-order dispersion coefficient $a_3$ determines the instability bandwidth and suppresses low-wavenumber perturbations, while the cubic nonlinearity $b_1$ sets the energy scale of the soliton. The parabolic (cubic–quintic) self-phase modulation modifies the effective nonlinear response at high intensities, leading to stabilization or destabilization depending on the sign and magnitude of $b_2$ . These mechanisms are directly relevant to photonic crystal fibers and highly nonlinear waveguides, where higher-order dispersion and non-Kerr nonlinearities are experimentally accessible. Collectively, these findings highlight the delicate interplay among key physical mechanisms that sustain stable localized structures in perturbed nonlinear media. The stability analysis, based on linear perturbation Theory confirms that the obtained soliton solutions remain robust under small perturbations when the essential parametric constraints are satisfied. These constraints, particularly the balance condition among perturbation coefficients and the compatibility relations for the soliton velocity is critical for ensuring the existence, structural integrity, and long-term propagation of the solitons. The key tunable parameters governing solution behavior are the fourth-order dispersion coefficient $a_3$, which controls soliton width and the modulational instability bandwidth in dispersion-engineered photonic crystal fibers, and the cubic nonlinearity coefficient $b_1$, which governs soliton amplitude and energy. The linear stability analysis yields an explicit eigenvalue dispersion relation and establishes a critical wavenumber threshold beyond which modulational Instability sets in, providing a quantitative stability boundary for practical optical system design.

Overall, the results enrich the theoretical understanding of perturbed nonlinear Schrödinger-type models and underscore their importance for applications in high-speed optical communication systems, ultrafast signal processing, and related fields of nonlinear optics and photonics. The bright and dark soliton solutions are of immediate Relevance to soliton-based transmission and dark-pulse modulation formats, while the Weierstrass elliptic solutions offer a theoretical framework for understanding periodic wave structures in actively mode-locked fiber lasers. The established parametric constraints translate directly into design conditions on the fiber dispersion profile and the nonlinear coefficient, supporting a practical system optimization.

The principal novelty of this work is the simultaneous unified treatment of parabolic-law nonlinearity, fourth-order dispersion, and multi-term perturbative effects within a single algebraic framework, yielding exact solution families, including Weierstrass elliptic solutions, not previously reported for this model. A comparative analysis demonstrates that IMETFM subsumes classical methods, including the tanh-function method, the $G'/G$-expansion, and the modified Kudryashov approach as limiting cases of its five-parameter auxiliary equation, achieving strictly greater solution diversity without requiring integrability. The findings pave the way for future work, including more detailed numerical simulations, experimental validations in optical fiber setups, extensions to higher-dimensional systems, and further exploration of soliton interactions and control mechanisms. Additional future directions include nonlinear and Lyapunov stability analyses to assess long-time robustness; extension of the framework for variable-coefficient, stochastic, and fractional-order NLSE models; exploration of $(2{+}1)$-dimensional soliton dynamics; investigation of multi-soliton interactions and bound-state formation; and experimental validation in photonic crystal fiber platforms, where $a_3$ and $b_1$ are independently tunable.

## Data Availability

The datasets used and/or analysed during the current study are available from the corresponding author on reasonable request.
